# Transumbilical single-incision laparoscopic distal pancreatectomy: preliminary experience and comparison to conventional multi-port laparoscopic surgery

**DOI:** 10.1186/1471-2482-14-105

**Published:** 2014-12-10

**Authors:** Dianbo Yao, Shuodong Wu, Yongnan Li, Yongsheng Chen, Xiaopeng Yu, Jinyan Han

**Affiliations:** Department of General Surgery, Shengjing Hospital, China Medical University, Shenyang, 110004 China

**Keywords:** Single-incision laparoscopic surgery, Distal pancreatectomy, Minimally invasive surgery, Multi-incision laparoscopic surgery

## Abstract

**Background:**

Single-incision laparoscopic surgery (SILS), which has been demonstrated to be safely applied on kinds of surgeries, may represent an improvement over conventional multi-port laparoscopic surgery. However, there are still few clinical experiences of SILS in pancreatic surgery until now. In this study, we will summarize our experience of transumbilical single-incision laparoscopic distal pancreatectomy (TUSI-LDP), and compare its related parameters with conventional multi-port laparoscopic distal pancreatectomy (C-LDP).

**Methods:**

A retrospective analysis was conducted for the patients who underwent C-LDP or TUSI-LDP in our department. The demographic data, operative parameters, and postoperative complications in the two groups were summarized and compared.

**Results:**

Laparoscopic distal pancreatectomy was performed in a total of 21 cases, among which TUSI-LDP was performed in 14 cases. As far as the demographical results concerned, there were no significant differences between the two groups. The conversion to open surgery was conducted in one case in the TUSI-LDP group because of severe adhesion between pancreatic cyst and surrounding tissues, while in the C-LDP group the only one conversion was for the difficult detection of small lesion. The mean operating time and intraoperative blood loss in TUSI-LDP group was a little shorter (166.4 ± 57.4 versus 202.1 ± 122.5 minutes, p > 0.05, and 157.1 ± 162.4 versus 168.6 ± 157.4 ml, p > 0.05). The postoperative pain and post-operation lengths of hospital stay in the TUSI-LDP group were also less, though there was no significant statistical difference between the two groups. For the post-operation complications, in TUSI-LDP group the pancreatic leakage occurred in only one case, and ceased spontaneously with only a drain for 61 days. There were no other complications including postoperative hemorrhage, venous thrombosis, infections and so on in both groups.

**Conclusion:**

For the experienced laparoscopic surgeons, in selected patients, TUSI-LDP is a feasible technique, with excellent cosmetic effect, less postoperative pain and post-operation lengths of hospital stay. With the experience accumulated, the operating time and intraoperative blood loss of TUSI-LDP could also gradually reduce.

## Background

Currently, the application of laparoscopic surgery for the distal pancreatectomy seems to become a trend in surgical technique, and might be considered as the first approach for distal pancreatectomy in the near future, possibly owing to its clear visual field, less injury, less postoperative pain, better cosmetic results, and faster recovery of patients [1–3]. Recently, for further minimizing surgical trauma by reducing the number of the port, many experienced laparoscopic surgeons have tried to develop a new minimally invasive technique called “single incision laparoscopic surgery” (SILS), which has been now successfully and widely applied in many fields of abdominal surgery [4, 5]. However, The SILS performed on the pancreatic lesions has been reported only recently, and the experience is still limited now [6–13]. Therefore, more studies are still required to confirm the feasibility of transumbilical single-incision laparoscopic distal pancreatectomy (TUSI-LDP). In addition, there have been few comparisons with standard laparoscopic distal pancreatectomy in the literature until now.

In this study, we would report 14 cases in which TUSI-LDP was performed in the Shengjing Hospital of China Medical University, to summarize the clinical experiences, and the related data was also compared with that of conventional multi-port laparoscopic distal pancreatectomy (C-LDP).

## Methods

### Patient selection and data collection

The criteria for patient selection in our department for laparoscopic distal pancreatectomy are as follows: a benign lesion was found in preoperative examination, and distal pancreatectomy was intented to be performed. The diameter of the lesion should be less than 3.5 cm, and could be a little larger for a cystic lesion. The patient has strong preference for cosmetic appearance, with no contraindications for laparoscopic surgery. All the patients participating in this study gave informed consent, for SILS operations and the publication of their individual clinical details. The patient records were also granted by the hospital to be accessed. This study was approved by the ethical committee of Shengjing Hospital, China Medical University.

Since 2009, all the cases in which laparoscopic distal pancreatectomy was performed in our department in the Shengjing Hospital of China Medical University were retrospectively reviewed. Medical records were reviewed to collect relevant information in the perioperative period. Operation records were reviewed to obtain operation indications, incision length, operative time, estimated blood loss, intraoperative complications and so on. Pathology reports were reviewed to obtain final diagnosis. Medication records were reviewed to determine analgetica used during the hospital stay. Daily progress notes were reviewed to document length of stay and perioperative complications, and follow-up by telephone was for postoperative complications within 30 days.

### Surgical technique

**Figure 1:**
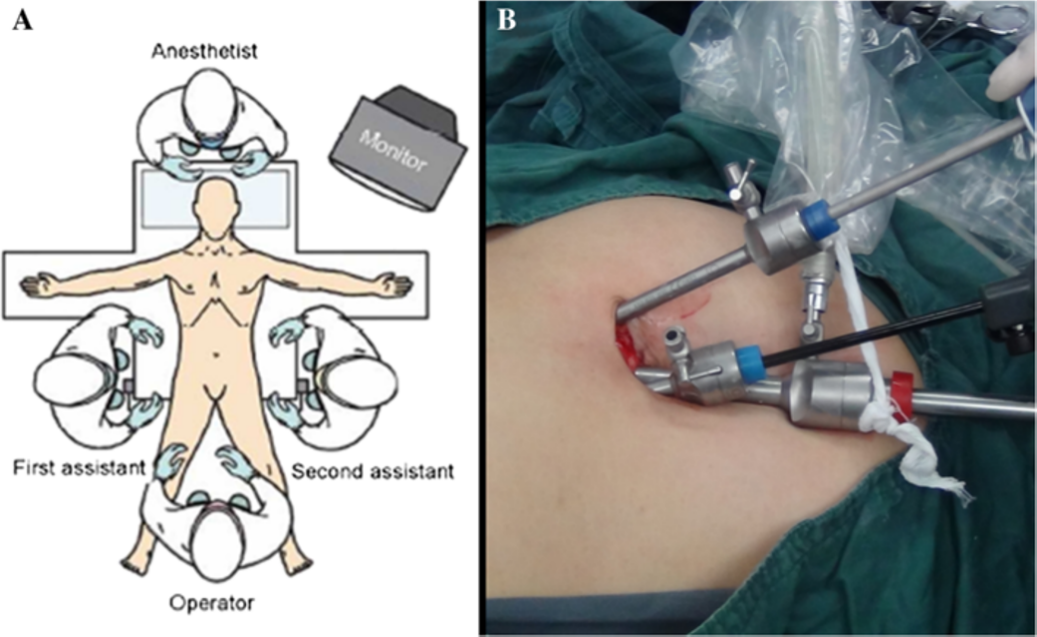
**Operating room setting and access site in the umbilicus. (A)** Operating room setting: the operator, assistants and monitor. **(B)** Access site in the umbilicus: the two trocars for the lens, grasper, and the plastic disposable trocar, for the ultrasonic scalpel.

In the TUSI-LDP group, after the induction of general endotracheal anesthesia, the patient was placed in a supine position, with legs apart (Figure 1A). A transumbilical 3 cm superficial longitudinal incision was made. After the maintenance of pneumoperitoneum, a 10 mm trocar was inserted into the lower margin of the incision, for the lens, and another two trocars were inserted on the superior margin of incision, the left one, 5 mm for the grasper, and the right one, 12 mm in diameter for the plastic disposable trocar (Figure 1B, case 12). In the MPLDP group, the incision at the umbilicus was 10 mm for the lens, and three additional trocars were used, one in the midline between the xiphoid process and the umbilicus, one lateral to the right rectus muscle at the level of the umbilicus and the other one subcostally in the medioclavicular line.

**Figure 2:**
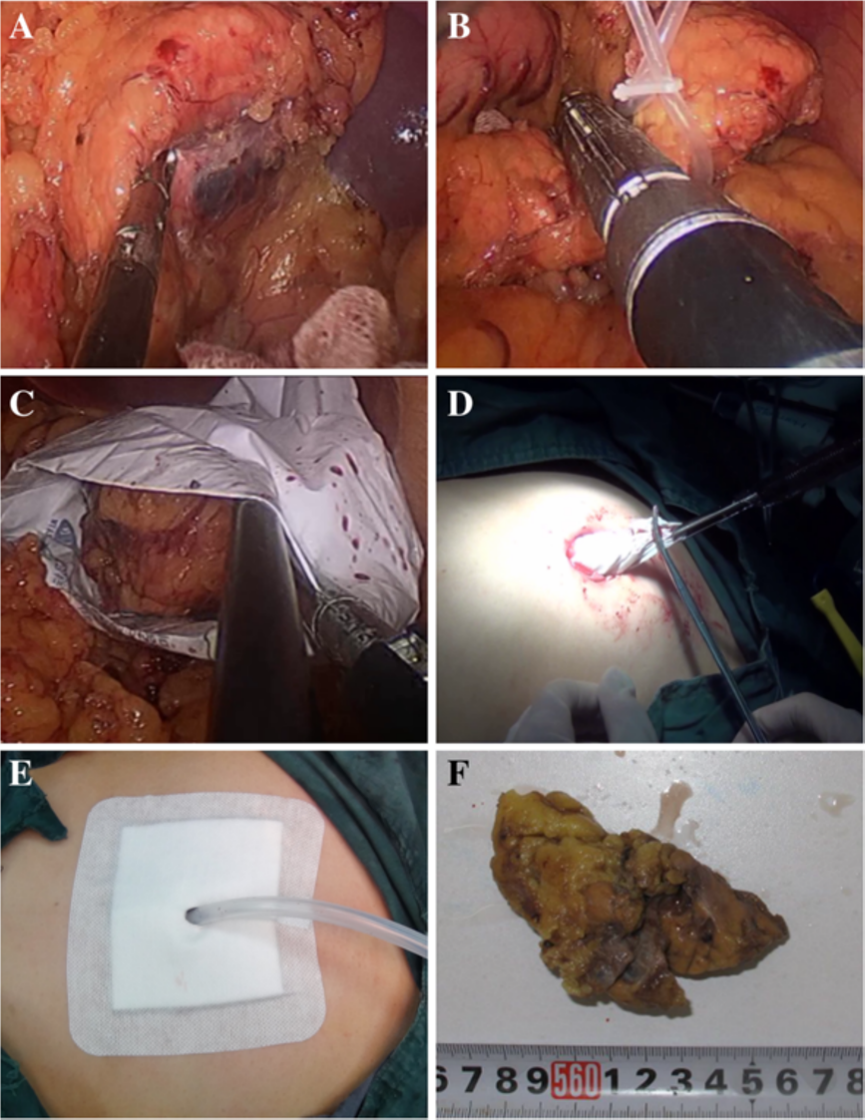
**Operative procedure of laparoscopic distal pancreatectomy. (A)** Exposure of pancreas. The lesion is seen at the right side of the picture. **(B)** The body to tail of the pancreas is mobilized and lifted from the retroperitoneum by a cloth tape, and pancreatic body is transected with an endoscopic linear stapler device. **(C)** The lesion was put into a retrieval bag. **(D)** The cyst contents were aspirated within the retrieval bag. **(E)** The closed drains brought out through the transumbilical incision. **(F)** The lesion, the serous cystadenoma in pathologic diagnosis.

The operative procedure was similar in the two groups. The operation began with the division of the gastrocolic ligament and the lower part of the gastrolienal ligament, to expose the body and tail of the pancreas and confirm the location and range of the mass or cyst (Figure 2A, case 12). Then, the serous membrane was dissected along the lower edge of pancreas on the right of the lesion. Loose connective tissue between the dorsal surface of the pancreas and the posterior abdominal wall was dissociated carefully, to not injure the splenic artery or splenic vein. When a tunnel would be built at the posterior surface of normal pancreatic body, the grasper was inserted into the tunnel and carried forward, to enlarge the visual fields behind the pancreas. The loose connective tissue was dissociated carefully by the ultrasonic scalpel towards the tail of the pancreas, and then the mass in the body of the pancreas was lifted and separated from posterior abdominal wall. When the body and tail of the pancreas from the normal pancreatic tissue on the right of the mass to the tail of pancreas near the hilum of spleen was completely separated, the pancreas would be divided with an endoscopic linear stapler device (Figure 2B).

If a splenectomy was to be simultaneously performed, the superior part of the gastrosplenic ligament, splenophrenic ligament, splenogastric ligament should be respectively dissected, and then the spleen was removed with the pancreas specimen. In the cases among which a spleen-preserving laparoscopic distal pancreatectomy was to be performed, the splenic vessels were mostly divided, with the short gastric vessels preserved to provide a blood supply. In some cases, the pancreatic branches of splenic vessels could be well dissected and divided, and the splenic vessels could also be separated from the pancreas and preserved.

A retrieval bag was placed through the plastic disposable trocar. The specimen was wrapped in the retrieval bag (Figure 2C) and extracted from the umbilical incision (Figure 2D). A closed drain was then placed near the stump of the pancreas, and brought out through the transumbilical incision (Figure 2E). A purse-string suture was beforehand reserved surrounding the drainage tube, and could be tensed to close the hole at the umbilicus when the intrabdominal drainage tube was pulled out.

### Postoperative treatment

Patients were transferred to the recovery room after surgery. Nasogastric tube was removed after return of intestinal function, and oral feeding was mostly initiated immediately, usually on the fourth postoperative day. As a closed drainage was placed near the stump of the pancreas, the content and volume of pancreatic amylase, and bleeding, was closely monitored. For slight pancreatic leakage, the remaining drainage time was extended, and the regulation of food and use of somatostatin was utilized for healing. The drainage tube could be pulled out when the drainage volume was less than 10 ml in 24 hours and there were no biochemical or clinical signs of a pancreatic fistula. Patients were discharged when tolerating a soft diet and no signs of complication were identified, and a strict follow-up was still made.

### Statistical analysis

Continuous data are presented as the mean ± standard deviation and the range. Categorical variables are expressed as numbers and percentages for each group. Continuous variables were compared between the two groups using an unpaired-sample student *t* test and Mann–Whitney test. Results were considered statistically significant for p < 0.05.

## Results

Since 2009, laparoscopic distal pancreatectomy was performed in a total of 21 cases, among which TUSI-LDP was performed in 14 cases. In the TUSI-LDP group, the characteristics of patients are listed in Table 1. Only one patient of the 14 cases was male, and the age ranged from 20 to 73 years old, with the average of 40.2 years old. The average of body mass index (BMI) is 22.6 (18.4 ~ 27.0). Among the 14 cases, 6 cases were diagnosed as pancreatic mucinous cystadenoma, 3 cases were diagnosed as the pancreatic cyst, 2 cases were diagnosed as splenic artery aneurysm, and the other 3 cases were diagnosed as pancreatic serous cystadenoma (Figure 2F), islet cell tumor and abdominal cavity fibromatosis involving the tail of pancreas respectively. In the C-LDP group, only one patient of the 7 cases was male. The average age was 50.4 ± 11.3 years old (range, 35–65), and the average of BMI is 23.3 (21.3 ~ 25.2). The related parameters in the C-LDP group are evaluated as a control group.

**Table 1:** **General information of 14 cases**

Patient number	Diagnosis	Tumor location	Age	Sex	BMI	Maximum diameter of lesion
1	Mucinous cystadenoma	body of pancreas	46	F	24.6	5
2	Fibromatosis	abdominal cavity	20	F	18.6	3.5
3	Serositic cyst	tail of pancreas	36	F	23.4	4.5
4	Pancreatic cyst	body of pancreas	42	F	22.2	4.5
5	Artery aneurysm	spleen	22	F	21.6	3.5
6	Mucinous cystadenoma and SPT	body and tail of pancreas	34	F	22.8	3.5
7	Pancreatic cyst	tail of pancreas	34	F	23.4	3.5
8	Mucinous cystadenoma	body and tail of pancreas	39	F	22.6	3
9	Islet cell tumor	tail of pancreas	73	F	26.2	1.2
10	Mucinous cystadenoma	body and tail of pancreas	27	F	22.2	4
11	Mucinous cystadenoma	tail of pancreas	45	F	21.8	6.2
12	Serous cystadenoma	tail of pancreas	50	F	21.5	3.2
13	Mucinous cystadenoma	tail of pancreas	37	F	18.4	11
14	Artery aneurysm	spleen	58	M	27.0	3.8

BMI body mass index; SPT solid-pseudopapillary tumor.

Operative data are given in Table 2. The TUSI-LDP were successfully performed in the 13 cases, while in the other case the operation conversed to conventional open surgery due to severe adhesion between pancreatic cyst and surrounding tissues. Among these 14 cases, laparoscopic distal pancreatectomy with splenectomy was performed in 7 cases (case 1, 3, 4, 5, 10, 11, 14), while spleen-preserving laparoscopic distal pancreatectomy was performed in the other 7 cases, including 2 cases in which the splenic vessels were successfully preserved (Table 2). The median operative time was (166.4 ± 57.4) min, with all procedures finished in <240 min in the 13 cases with successful TUSI-LDP, and the operative time in the case with conversion to open surgery was 300 min. The median estimated intraoperative blood loss was (157.1 ± 162.4) ml (10–500 ml), with none of the patients requiring perioperative transfusion of red blood cells. As for the postoperative complications, only in one case with the diagnosis of the pancreatic cyst, the pancreatic leakage occurred, and ceased spontaneously with only a drain for 61 days. There were no other complications including postoperative hemorrhage, venous thrombosis, fever, infection and so on. Postoperative umbilical incision healed well, with no obvious scar, and cosmetic result was well. The patients were discharged from hospital in a mean of (7.6 ± 1.4) d (range, 5 to 10 d), with no mortality. All the patients resumed daily activities quickly.

**Table 2:** **Operative parameters and postoperative recovery of patients**

Cases	Operation time (min)	Intraoperative blood loss (ml)	Conversion to multi-incision surgery	Postoperative evacuating time (day)	Food intake time (day)	Drainage time (day)	Hospital stay (day)	Postoperative hemorrhage	Pancreatic leakage
1	300	500	yes	3	3	4	8	no	no
2^*^	240	500	no	4	4	7	9	no	no
3	150	10	no	3	3	>7	7	no	yes
4	125	100	no	4	5	7	8	no	no
5	170	10	no	3	3	4	6	no	no
6^*^	110	30	no	3	3	4	6	no	no
7^*^	115	50	no	2	3	7	8	no	no
8^*^	165	100	no	5	5	10	10	no	no
9^#^	170	50	no	3	4	7	8	no	no
10	155	200	no	4	5	7	7	no	no
11	95	200	no	3	4	5	5	no	no
12^#^	120	50	no	3	4	6	7	no	no
13^*^	185	200	no	3	4	7	9	no	no
14	230	200	no	5	5	8	9	no	no

*Splenic preservation; ^#^Splenic and its vessels preservation.

For preoperative characteristics, there were no statistically significant differences between the two groups (Table 3). For the related data of operation (Table 4), the conversion to open surgery was conducted in one case in the TUSI-LDP group because of severe adhesion between pancreatic cyst and surrounding tissues, while in the C-LDP group the only one conversion was for the difficult detection of small lesion. The mean operative time was a little shorter in the single-incision laparoscopic group (166.4 ± 57.4 versus 202.1 ± 122.5 min, p > 0.05), and the mean estimated blood loss in the single-incision group was also a little smaller (157.1 ± 162.4 versus 168.6 ± 157.4 ml, p > 0.05), though there was no significant difference between the two groups. There were no requiring blood transfusions in the two groups, and no deaths in either group. As for the postoperative complications, in only one case in the single-incision group the pancreatic leakage occurred, and ceased spontaneously with only a drain for 61 days. The use of in-hospital postoperative narcotics was evaluated in both groups of patients. While the patients in the TUSI-LDP group (0.7 ± 0.6 times) used a lower total dose of narcotic medication than that in the C-LDP group (1.1 ± 0.7 times), the difference was not significant, either. The mean length of stay in the two groups was 7.6 days and 9.0 days respectively.

**Table 3:** **Demographical characteristics of the patients**

	SILS (n =14)	Conventional (n = 7)	P
Age, mean ± SD [range]	40.2 ± 14.1 [20–73]	50.4 ± 11.3 [35–65]	0.66
Sex (% men)	7.1	14.3	0.61
Weight, mean ± SD [range]	59.6 ± 8.9 [45–80]	60.4 ± 5.1 [54–67]	0.21
BMI, mean ± SD [range], kg/m2	22.6 ± 2.4 [18.4–27.0]	23.3 ± 1.3 [21.3–25.2]	0.29
Size of lesion,, mean ± SD [range], cm	4.3 ± 2.2 [1.2–11]	3.7 ± 2.2 [0.7–6.0]	0.47
Lesion type (benign/malignant)	0/14	1/6	0.16

**Table 4:** **Operative and postoperative results**

	SILS (n =14)	Conventional (n = 7)	P
Operating time, mean ± SD, min	166.4 ± 57.4	202.1 ± 122.5	0.15
Estimated blood loss,mean ± SD, ml	157.1 ± 162.4	168.6 ± 157.4	0.66
Scale of pain, mean ± SD	0.7 ± 0.6	1.1 ± 0.7	0.90
Conversion to open surgery, %	1/14	1/7	0.61
Complications, %	1/14	0/7	0.48
Length of hospital stay, mean ± SD, d	7.6 ± 1.4	9.0 ± 3.0	0.17

## Discussion

In recent years, the search for less morbidity and greater patient comfort has led surgeons to develop newer means of access to the abdominal cavity with less surgical trauma, such as natural-orifice transluminal endoscopic surgery and single-incision laparoscopic surgery. The scarce reproducibility and difficulty involved with the natural orifice technique made most surgeons opt for the single-incision technique, for its similarity with conventional laparoscopy and lower requirement of specific equipments. Since the first documented single incision laparoscopic procedure in 1997, SILS has already been applied dramatically in many surgical procedures, such as cholecystectomy [4], appendectomy [5, 14], total extraperitoneal inguinal hernia repair [15], sleeve gastrectomy [16], gastrojejunostomy [17], splenectomy [18], nephrectomy [19], liver resection [20], and so on. However, TUSI-LDP has still been rarely reported, possibly because pancreatic surgery represents one of the most challenging areas in digestive surgery.

In 2010, Barbaros U et al. first reported the TUSI-LDP with splenectomy, and it was described that the overall procedures were similar to that performed in the conventional multi-port laparoscopic pancreatectomy [6]. The operation was successfully finished even though in the retroperitoneal region there was dense fibrosis caused by a previous left nephrectomy, confirming that TUSI-LDP could be performed technically. Since then, TUSI-LDP were reported a total of 26 cases, among which, the largest study was reported by us [11]. Recently, three more cases were performed in our department. Now, during the 29 cases, transumbilical single-incision laparoscopy spleen-preserving distal pancreatectomy in 16 cases and single-incision laparoscopy distal pancreatectomy without splenic preservation in 13 case were performed, and the patients’ postoperative recoveries were all uneventful [6–13]. Now, these experiences well confirm the feasibility and safety of the TUSI-LDP in selected cases. However, for the pancreatic surgery, comparative studies are still needed, to compare the related parameters of the single-incision and conventional laparoscopic techniques, and lay down the foundation for the possible indications for this type of access.

As far as operating time concerned, some published studies for SILS reveal a longer operating time than conventional laparoscopy [21–23]. As in SILS all instruments are closely packed together, and the instruments, which are limited within a small range of motion, would interfere with each other, the operation of SILS is though to be more difficult. These would increase the difficulty of learning and practice, and also increase the operating time. However, as the SILS were performed, the operation could be more and more smoothly. One study [24], comparing colon resections for cancer using the 2 techniques, reported no differences and operating times were practically the same, although the size of the series was small. Now, in our study, we observed that in uncomplicated distal pancreatectomy with normal characteristics, operating times in SILS group would be similar or even shorter than that in conventional laparoscopy group, mainly because our experiences for LDP and SILS had accumulated much before TUSI-LDP began to be performed, making the operations in TUSI-LDP much more smoothly, just as our results for the gastric GIST [25]. Similarly in our study, the intraoperative blood loss in the single-incision laparoscopic group was also smaller than that in the conventional laparoscopic group, though the difference did not reach statistical significance. As our experiences of TUSI-LDP gradually accumulated, we could be more careful for the vessels during operation, and intraoperative blood loss could also be gradually well controlled. However, operating time or intraoperative blood loss might be much longer when there was severe adhesion between pancreatic cyst and surrounding tissues. In this study, one patient required even a conversion to open surgery because of severe adhesion.

Many studies have suggested that the single-incision laparoscopic surgery approach may have some advantages over conventional laparoscopic surgery: greater patient comfort, less postoperative pain, and a better cosmetic outcome due to a scareless procedure [26]. However, for the pancreatic surgery, the related reports were lacking. The less injury and less postoperative pain of SILS might be related with reducing the size of the skin incision and not perforating the aponeurosis or muscle. In some complex surgical procedures, such as colectomy, which requires a greater number of incisions or even minilaparotomy to complete the operation, postoperative pain may have important clinical repercussions in satisfaction, quality of life, and health state. Some previous prospective studies show no differences for the postoperative pain [21, 22, 27, 28], but the results obtained in our study indicated that although there were no significant difference, the patients in the TUSI-LDP group indeed used a lower total dose of narcotic medication. Therefore, the controversy for the postoperative pain still exists, waiting for further confirmed.

For the postoperative complications, the well visual fields of operation provided by the laparoscope, and the rational utilization of sealing devices have made the complication in laparoscopic surgery rarely occur. It was found that compared with open distal pancreatectomy, LDP patients had significant fewer complications [29]. Cho CS et al. explored the risk factors for pancreatic fistula after distal pancreatectomy, and found that preoperative characteristics may identify cohorts of patients who will benefit more from LDP, and no patient cohorts had higher postoperative complication rates after LDP than open distal pancreatectomy, suggesting that LDP may be the better operative procedure of choice for less pancreatic fistula or other complications [30]. Now, for the TUSI-LDP, the pancreatic fistula occurred in only one case of our 14 cases and one case in the English literature [6], and conservative treatment was effective in the both cases. There were no other complications including postoperative hemorrhage, venous thrombosis, fever, infection and so on. These results suggest the patients with TUSI-LDP may also benefit much for less postoperative complications. Certainly, more definitive prospective and randomized comparisons are still needed for further confirmation.

As for the post-operation lengths of hospital stay, though no significant difference was found between the two groups, the mean lengths of hospital stay in the SILS group were also a little shorter. It may be related with the reduced postoperation pain, and early recovery of oral feeding. Certainly, we are beginning to try applying a relative fast-track protocol in one patient (case 11) with uncomplicated pancreatic cyst without increasing the number of complications, and the oral feeding initiation and the discharge time could still be futher earlier [31] in the near future.

Today, SILS is becoming popular, and its purpose is to cure the disease in a cosmetic method with minimal invasion. As these results suggest, the single incision approach could be applied successfully in the pancreatic surgery, providing high degree of satisfaction and well cosmetic advantages, though much more techniques are needed for the operator. It does not increase the rate of complications and represents a possible alternative to conventional laparoscopic distal pancreatectomy. With the related experience acquired, we believe that it could be applied more and more widely [32].

## Conclusions

Our study suggests that for the experienced laparoscopic surgeons, TUSI-LDP is feasible and safe, with excellent cosmetic effect, and the single-incision technique is comparable to standard laparoscopic distal pancreatectomy in terms of operative time and perioperative outcomes. Certainly, the advantages and disadvantages of the TUSI-LDP compared with the conventional LDP still need further evaluated in prospective clinical researches.

### Consent

Written informed consent was obtained from all the patients for publication of related data and any accompanying images. A copy of the written consent is available for review by the Editor-in-Chief of this journal.

## Abbreviations

SILS: Single-incision laparoscopic surgery
TUSI-LDP: Transumbilical single-incision laparoscopic distal pancreatectomy
C-LDP: Conventional multi-port laparoscopic distal pancreatectomy
BMI: Body mass index.

## References

[1] Lee SY, Allen PJ, Sadot E, D'Angelica MI, DeMatteo RP, Fong Y, Jarnagin WR, Kingham TP (2014). Distal Pancreatectomy: A Single Institution's
Experience in Open, Laparoscopic, and Robotic Approaches. J Am Coll Surg.

[2] Røsok BI, Marangos IP, Kazaryan AM, Rosseland AR, Buanes T, Mathisen O, Edwin B (2010). Single-centre experience of laparoscopic pancreatic
surgery. Br J Surg.

[3] Kneuertz PJ, Patel SH, Chu CK, Fisher SB, Maithel SK, Sarmiento JM, Weber SM, Staley CA, Kooby DA (2012). Laparoscopic distal pancreatectomy: trends and lessons
learned through an 11-year experience. J Am Coll Surg.

[4] Tranchart H, Ketoff S, Lainas P, Pourcher G, Di Giuro G, Tzanis D, Ferretti S, Dautruche A, Devaquet N, Dagher I (2013). Single incision laparoscopic cholecystectomy: for what
benefit?. HPB (Oxford).

[5] Frutos MD, Abrisqueta J, Lujan J, Abellan I, Parrilla P (2013). Randomized prospective study to compare laparoscopic
appendectomy versus umbilical single-incision appendectomy. Ann Surg.

[6] Barbaros U, Sümer A, Demirel T, Karakullukçu N, Batman B, Içscan Y, Sarıçam G, Serin K, Loh WL, Dinççağ A, Mercan S (2010). Single incision laparoscopic pancreas resection for
pancreatic metastasis of renal cell carcinoma. JSLS.

[7] Kuroki T, Adachi T, Okamoto T, Kanematsu T (2011). Single-incision laparoscopic distal
pancreatectomy. Hepatogastroenterology.

[8] Chang SK, Lomanto D, Mayasari M (2012). Single-port laparoscopic spleen preserving distal
pancreatectomy. Minim Invasive Surg.

[9] Misawa T, Ito R, Futagawa Y, Fujiwara Y, Kitamura H, Tsutsui N, Shiba H, Wakiyama S, Ishida Y, Yanaga K (2012). Single-incision laparoscopic distal pancreatectomy
with or without splenic preservation: how we do it. Asian J Endosc Surg.

[10] Srikanth G, Shetty N, Dubey D (2013). Single incision laparoscopic distal pancreatectomy
with splenectomy for neuroendocrine tumor of the tail of pancreas. J Minim Access Surg.

[11] Yao D, Wu S, Tian Y, Fan Y, Kong J, Li Y (2013). Transumbilical Single-Incision Laparoscopic Distal
Pancreatectomy: Primary Experience and Review of the English
Literature. World J Surg.

[12] Haugvik SP, Røsok BI, Waage A, Mathisen O, Edwin B (2013). Single-incision versus conventional laparoscopic
distal pancreatectomy: a single-institution case–control study. Langenbecks Arch Surg.

[13] Machado MA, Surjan RC, Makdissi FF (2013). First single-port laparoscopic pancreatectomy in
Brazil. Arq Gastroenterol.

[14] Hong TH, Kim HL, Lee YS, Kim JJ, Lee KH, You YK, Oh SJ, Park SM (2009). Transumbilical single-port laparoscopic appendectomy
(TUSPLA): scarless intracorporeal appendectomy. J Laparoendosc Adv Surg Tech A.

[15] Filipovic-Cugura J, Kirac I, Kulis T, Jankovic J, Bekavac-Beslin M (2009). Single-incision laparoscopic surgery (SILS) for
totally extraperitoneal (TEP) inguinal hernia repair: first case. Surg Endosc.

[16] Reavis KM, Hinojosa MW, Smith BR, Nguyen NT (2008). Single-laparoscopic incision transabdominal surgery
sleeve gastrectomy. Obes Surg.

[17] Bucher P, Pugin F, Morel P (2009). Transumbilical single-incision laparoscopic
intracorporeal anastomosis for gastrojejunostomy: case report. Surg Endosc.

[18] Barbaros U, Dinc¸c¸ag˘ A (2009). Single incision laparoscopic splenectomy: the first
two cases. J Gastrointest Surg.

[19] Raman JD, Bagrodia A, Cadeddu JA (2008). Single-incision, umbilical laparoscopic versus
conventional laparoscopic nephrectomy: a comparison of perioperative outcomes
and short-term measures of convalescence. Eur Urol.

[20] Zhao G, Hu M, Liu R, Xu D, Ouyang C, Xu Y, Jiao H, Wang B, Gu X (2011). Laparoendoscopic single-site liver resection: a
preliminary report of 12 cases. Surg Endosc.

[21] Vidal O, Valentini M, Ginest’a C, Martí J, Espert JJ, Benarroch G, García-Valdecasas JC (2010). Laparoendoscopic single-site surgery
appendectomy. Surg Endosc.

[22] Lee J, Baek J, Kim W (2010). Laparoscopic transumbilical single-port appendectomy:
initial experience and comparison with three-port appendectomy. Surg Laparosc Endosc Percutan Technol.

[23] St Peter SD, Adibe OO, Juang D, Sharp SW, Garey CL, Laituri CA, Murphy JP, Andrews WS, Sharp RJ, Snyder CL, Holcomb GW, Ostlie DJ (2011). Single incision versus standard 3-port laparoscopic
appendectomy: a prospective randomized trial. Ann Surg.

[24] Papaconstantinou HT, Thomas JS (2011). Single-incision laparoscopic colectomy for cancer:
assessment of oncologic resection and short-term outcomes in a case-matched
comparison with standard laparoscopy. Surgery.

[25] Kong J, Wu SD, Su Y, Fan Y (2014). Single incision versus conventional laparoscopic
resection in gastrointestinal stromal tumors: a retrospective cohort analysis at
a single tertiary care center. Onco Targets Ther.

[26] Ahmed K, Wang TT, Patel VM, Nagpal K, Clark J, Ali M, Deeba S, Ashrafian H, Darzi A, Athanasiou T, Paraskeva P (2011). The role of single-incision laparoscopic surgery in
abdominal and pelvic surgery: a systematic review. Surg Endosc.

[27] Raakow R, Jacob DA (2011). Initial experience in laparoscopic single-port
appendectomy: a pilot study. Dig Surg.

[28] Teoh AY, Chiu PW, Wong TC, Wong SK, Lai PB, Ng EK (2011). A case-controlled comparison of singlesite access
versus conventional three-port laparoscopic appendectomy. Surg Endosc.

[29] Kooby DA, Gillespie T, Bentrem D, Nakeeb A, Schmidt MC, Merchant NB, Parikh AA, Martin RC, Scoggins CR, Ahmad S, Kim HJ, Park J, Johnston F, Strouch MJ, Menze A, Rymer J, McClaine R, Strasberg SM, Talamonti MS, Staley CA, McMasters KM, Lowy AM, Byrd-Sellers J, Wood WC, Hawkins WG (2008). Left-sided pancreatectomy: a multicenter comparison of
laparoscopic and open approaches. Ann Surg.

[30] Cho CS, Kooby DA, Schmidt CM, Nakeeb A, Bentrem DJ, Merchant NB, Parikh AA, Martin RC, Scoggins CR, Ahmad SA, Kim HJ, Hamilton N, Hawkins WG, Weber SM (2011). Laparoscopic versus open left pancreatectomy: can
preoperative factors indicate the safer technique?. Ann Surg.

[31] Elola-Olaso AM, Allen A, Gagliardi RJ (2009). Laparoscopic distal pancreatectomy for solid and
cystic pancreatic neoplasms: outpatient postoperative management. Surg Laparosc Endosc Percutan Tech.

[32] Tsai AY, Selzer DJ (2010). Single-port laparoscopic surgery. Adv Surg.

[CR33] The pre-publication history for this paper can be accessed here:http://www.biomedcentral.com/1471-2482/14/105/prepub

